# Colon and rectal cancer incidence and water trihalomethane concentrations in New South Wales, Australia

**DOI:** 10.1186/1471-2407-14-445

**Published:** 2014-06-17

**Authors:** Md Bayzidur Rahman, Christine Cowie, Tim Driscoll, Richard J Summerhayes, Bruce K Armstrong, Mark S Clements

**Affiliations:** 1 School of Public Health and Community Medicine, UNSW Australia, Room 215, Samuels building (F25), Botany Street, Randwick NSW 2052, Australia; 2 Woolcock Institute of Medical Research, The University of Sydney, Sydney, Australia; 3 South Western Sydney Clinical School, UNSW, Sydney, Australia; 4 Sydney School of Public Health, The University of Sydney, Sydney, Australia; 5 School of Health and Human Sciences, Southern Cross University, Lismore, Australia; 6 National Centre for Epidemiology and Population Health, Australian National University, Canberra, Australia; 7 Karolinska Institutet, Stockholm, Sweden

**Keywords:** Cancer, Colon, Rectal, Disinfection by-products, Chlorination, Ecological studies

## Abstract

**Background:**

There is evidence, although inconsistent, that long term exposure to disinfection by products (DBPs) increases the risk of bowel cancer. No study has been conducted in Australia to examine this association and due to difference in the methods of disinfection the risk can vary across geographical regions and. This study was conducted to analyse the association of trihalomethanes (THMs) in water with colon and rectal cancer in NSW Australia.

**Methods:**

Average yearly concentrations of total and individual species of THMs were obtained for 50 local government areas (LGAs). Indirectly-standardized incidence rates of colon and rectal cancers in LGAs for the period 1995 to 2001 were regressed against mean THM concentrations lagged five years, adjusting for socioeconomic status, high risk drinking, smoking status, usual source of water and year of diagnosis, including local and global random effects within a Bayesian framework. The incidence rate ratios (IRRs) for an interquartile range (IQR) increase in THMs were estimated.

**Results:**

Using five year lag of exposure there was a positive association between bromoform concentration and CRC in men (IRR = 1.025, 95% CI 1.010, 1.040) but not in women (IRR = 1.003, 95% CI 0.987, 1.018). The association in men was mainly found in colon cancer with bromoform (IRR = 1.035, 95% CI 1.017, 1.053). There was no appreciable association of colorectal cancer with other species of THMs. Sensitivity analyses did not materially change the associations observed.

**Conclusion:**

A positive association was observed between colon cancer and water bromoform concentrations in men. Given the potential population impact of such an association, further research into the relationship between THMs, particularly brominated species, and colorectal cancer is warranted.

## Background

Epidemiological studies have reported increased risks of bladder, colorectal and renal cancer, and adverse reproductive and developmental outcomes in people exposed to chlorinated drinking water or chemical by-products of chlorination (disinfection by-products or DBPs), although not consistently [1-7]. These studies have usually used measurements of trihalomethanes (THMs, e.g., total THM, chloroform, bromoform etc.), a major sub-group of DBPs, as a measure of exposure.

A recent meta-analysis of case–control and cohort studies of the association of DBPs with colorectal cancer (CRC) found summary odds ratios (ORs) of 1.30 (95% CI 1.06, 1.59) for rectal cancer and 1.27 (95% CI: 1.08, 1.50) for colon cancer, comparing the highest exposure category with the lowest [8]. Many of the studies in the meta-analysis did not report the actual concentration of THM in the water. Weaknesses in exposure measurement and in the control of potential confounders in many of the studies make these findings uncertain. In addition, all the studies included in the meta-analysis were conducted in Europe or in North America. It has been revealed in a recent meta-analysis and pooled analysis that the method of disinfection varies importantly between North America and Europe which may contribute to the differences in the risk of cancer [2]. Therefore, the findings from studies conducted in other countries may not be applicable to Australia.

Due to limited generalizability of the previous studies, we have conducted an ecological study as a starting point to explore the association in Australia. In this study, we investigated whether the incidence of CRC is associated with THM concentrations in two water supply regions in New South Wales, Australia. All public water supply systems in these regions are served by chlorinated or chloraminated water.

## Methods

We undertook a spatial ecological study with local government area (LGA) by calendar year as the unit of analysis. The study covered two separate geographical areas: the Sydney Water Corporation (SWC) region, which supplies water to 47 LGAs and the Hunter Water Corporation (HWC) region which supplies water to 5 LGAs. For each of the LGAs we sought data on the incidence of colorectal cancer from 2001 to 2006 and estimates of THMs in water supplies from 1995 to 2001, to allow a five-year lag. Among the DBP species, only THMs were routinely measured in these supplies; they had been consistently measured since 1995 in the SWC region and since 1997 in the HWC region.

In the HWC water supply system, water is piped into reservoirs in six water supply zones and then distributed to homes located in nine water distribution zones that contain the sampling sites. The SWC water supply system has 14 delivery systems encompassing 33 distribution systems, which, in turn, encompass 180 reservoir zones that contain the sampling sites. A detailed description of the study areas, the water supply system, disinfection practices etc. is given elsewhere [9].

### Estimation of THM concentrations at LGA level

We obtained data on THM concentrations for all the monitored sampling sites by date of sample collection from the SWC and the HWC and used them to estimate yearly average THM concentrations for the LGAs from 1995 (1997 in HWC) to 2001. For HWC, we used data at the distribution zone level. Complete monthly THM data for HWC were available at this level. For SWC, we averaged monthly concentrations of total THMs and THM species for each year across all sampling sites at the distribution system level. At this level, 10% to 20% of monthly average values were missing for the years 1995 to 1999 and fewer than 10% for the years after 1999. We used THM data at the distribution system level as our ecological exposure measure because distribution systems are of similar size to LGAs and most of the disinfection and re-chlorination happens at this level (Additional file 1).

We adopted a cluster mean approach to impute missing THM data from SWC. If a distribution system value was missing for any month we estimated it by taking the value for that month from the delivery system in which the distribution system lay. This approach provided complete data from 2000 onwards and nearly complete data (1% missing) for 1998 and 1999. For the preceding years approximately 80% of values were still missing. For 1996 and 1997 we obtained complete coverage by taking the annual average of THM concentration at the distribution system level. For 1995, THM values were still missing for six (18%) distribution systems. This approach was used in a previously published paper on maternal exposure to trihalomethanes and the risk of small for gestational age births in the Sydney and Hunter regions [10].

There is no spatial alignment of LGA and distribution zone or system boundaries in either HWC or SWC. Some distribution systems supply water to two or more LGAs. Four distributions systems in SWC supplied water to one LGA only, other systems, on average, supplied to four LGAs (min 2, max 16). In the SWC region, 14 of the LGAs were served by only one distribution system. On average, LGAs are supplied by three distribution systems (min 2 max 8). To cope with these spatial anomalies we calculated area weighted average THM concentrations for LGAs from the distribution zones or systems they covered, where the weights were the proportions of the LGA area that they covered. We used ArcGIS (version 9.3) [11] to determine the area coverage of LGAs by the distribution systems and also estimated the standard deviations (SDs) of the weighted mean THMs. This process left missing means for only two LGAs (out of 47) in the SWC region, which we excluded from the analysis. This imputation method has been validated in another study where it did not affect the effect estimates of small for gestational age [10].

In the SWC region, 18.7% of bromoform concentration values fell below the detection limit (DL) of 1 μg/L (1995 to 1998), and 63.3% of values fell below the DL of 3 μg/L (1999 onwards). In the HWC region, the DL for bromoform was 1 μg/L for the whole study period and 51% of all observations were below this DL. For all other species of THMs approximately 1% of values were below the specified DL. We replaced values below the DL with a value that was two thirds of the DL [12]. We conducted a sensitivity analysis by using a different method to replace the values below the DL (Additional file 2).

### Cancer incidence data

We obtained de-identified unit record data for CRC incidence by sex, five-year age group, year of diagnosis and LGA of residence from the NSW Central Cancer Registry for the period 2000 to 2006. Since the CRC rate is very low for age groups below 35 years, the analysis was restricted to people aged 35 years and above. Several studies have examined the agreement between the Central Cancer Registry data and medical records for colorectal cancer. One study examined the agreement between stage of colorectal cancer at diagnosis and found a 70% agreement between the registry data and data recorded in a survey of treating doctors [13]. Another study reported high agreement between self-reported cancers in older women in the Longitudinal Study in Women’s Health and records in the Central Cancer Registry with sensitivity of 90% (95% CI: 78.6-95.6) and specificity 99.3% (95% CI: 98.9-99.5) for colorectal cancer [14].

### Area-level covariates

We included five covariates on socio-economic status, high risk drinking, smoking status, drinking water treatment at home and usual source of drinking water (tap, bottle, rainwater, well, other) at the LGA level. In 2003, 88% of metropolitan areas used the public water supply, 9% bottled water, 1.4% rainwater and 0.2% private bore or well water [15]. We obtained area level information on socio-economic status. Socio-Economic Indexes for Areas (SEIFA) is a set of indexes produced by the Australian Bureau of Statistics (ABS) after every Census [16]. The SEIFA utilizes relevant Census data on education, income, employment and housing to produce index scores that rank areas based on their relative socioeconomic advantage and disadvantage [17]. We used the index of relative socio-economic disadvantage (IRSD) as our indicator of socio-economic status [18,19]. We used the IRSD from the 2001 census for 2001 to 2003 and from the 2006 census for 2004 to 2006. The LGA level IRSD scores were estimated by taking the population-weighted average scores of the census collector districts (typically having populations of around 300 people) in each LGA. A low index value indicates high proportions of low-income families and people with low skilled occupations or without training [19]. The other four area level indicators were obtained from the 2002 Adult Health Survey conducted by the NSW Health Department and applied to the LGAs [15]. The proportion of high risk drinking was defined as one or more of the following: consuming alcohol every day, consuming an average of four if male or two if female standard drinks a day or consuming six if male or four if female standard drinks on any one occasion or day [15]. The proportion of current smokers, and the proportion of people who use public tap water as the usual source of drinking water, were also included in the final model. The proportion of people who treat their drinking water before consumption was not significant and not included in the model.

### Statistical analysis

To model the risk of CRC with exposure we used observed and expected numbers of cases of CRC by year and LGA. Expected numbers were calculated using age and sex specific rates of CRC in NSW. To include socio-economic status in the analytical model we measured inequality in terms of the relative index of inequality [20]. It was estimated by the exponential of the negative of the parameter for the IRSD quintile in the regression model.

We used the observed number of cases as the outcome variable and the log of the expected number as the offset in a Poission regression model. We adopted a full Bayesian hierarchical framework to fit the model including the other covariates (year of diagnosis of CRC, SES, smoking status, usual source of drinking water, high risk drinking and region of residence (SWC or HWC)) and estimated the area-specific and overall incidence rate ratio (IRR) of CRC for THM exposure. We adjusted for region because of different levels of urbanicity as well as the difference in water source (HWC water sources are river based and ground water from coastal aquifers, while SWC water is supplied by a number of large dams/reservoirs). We used a conditional auto-regressive model to adjust for spatial autocorrelation and possibly some unknown factors [21], which entailed the addition of global and spatial random effects to our model [22]. The Markov Chain Monte Carlo (MCMC) method was used to sample from the posterior probability distribution. To determine how the spatial random effects term affected the overall model fit and the estimates, the Bayesian hierarchical models were fitted with and without the spatial random effect.

Pure specification bias (within-area variability bias) due to aggregating a nonlinear individual-level model over the within-area distribution of covariates can severely bias risk estimates in ecological studies [23-25]. To control for this bias we also fitted a non-linear model including the variance term (whose regression parameter is the square of the regression parameter for the mean term divided by two). Since some of the area means did not have a variance (when they came from only one observation), we examined the effect of missing variances by fitting another model restricted to data sets that had non-missing variances.

We made the a priori assumption of a five year lag between exposure and outcome. To assess the sensitivity of the results to this assumption, we reanalysed the data by averaging the exposure data (but not the outcome data) across the calendar years (1995 to 2001). Since the HWC region has only five LGAs and its inclusion required that we restrict the analysis to three exposure and outcome years, we also compared models including and excluding the HWC region.

All model comparisons were made using the deviance information criterion (DIC) [26]. For each Bayesian hierarchical model two MCMC chains from different starting points were estimated to assure convergence. Depending on the complexity of the models, the first half (20,000) of the total iterations in each chain were removed (as “burn-ins”). Convergence of the models was monitored by visual examination of MCMC chains, autocorrelation plots and Gelman–Rubin statistic plots. Full Bayes hierarchical models were estimated using WinBUGS version 1.4.3 software using the GeoBUGS package, with scripting in R version 2.9.1 using R2WinBUGS.

We obtained the exposure data on disinfection by-products from the SWC and HWC upon obtaining their official approval. We obtained the cancer incidence data from the NSW central cancer registry upon their approval. The area level data on other demographic factors are publicly available and do not require permission from any authorities to use them.

To conduct this study Ethics approval was obtained from the NSW Population & Health services Research Ethics Committee (Ref: **2006/03/002**).

## Results

The indirectly age-standardized incidence rate of CRC for the period 2000 to 2006 for ages 35 years and over in the SWC region was 69.8 (95% CI 67.6, 72.0) per 100,000 in men and 55.9 (95% CI 54.1, 57.7) per 100,000 in women. The corresponding rates in the HWC region were 81.3 (95% CI 75.6, 87.0) in men and 60.4 (95% CI 55.3, 65.6) in women.

The means of the estimated concentrations of THMs at LGA level in the SWC and HWC regions for 1995 to 2001 are shown in Table 1. They were generally similar in the two regions. The ranges for all THM species were greater in the SWC region than in the HWC region. Detailed information on area specific and yearly concentrations of THM species in the two regions is available elsewhere [9].

**Table 1:** **Descriptive statistics for THM concentration (μg/L) in SWC and HWC water supplies, 1995 to 2001**^**1**^

**Region**	**Statistics**	**Chloroform**	**Bromoform**^**2**^	**BDCM**	**DBCM**	**TTHM**
Sydney water corporation (n = 45)	Mean	37.3	2.86	16.9	6.93	64.0
	SD	24.0	2.87	5.32	3.34	29.8
	Median	33.2	2	17.3	5.5	66.1
	IQR	22.9	1.2	5.6	3.1	30.1
	Range	8.5 to 201	0.67 to 17.8	6.66 to 45.0	0.67 to 27.7	20.5 to 234
Hunter water corporation (n = 5)^3^	Mean	42.7	1.76	14.7	7.25	66.4
	SD	13.1	1.11	5.26	6.15	11.4
	Median	39.8	2.0	17.3	5.5	66.4
	IQR	22.2	1.3	5.6	3.2	29.0
	Range	23.3 to 65.0	0.67 to 3.95	5.64 to 24.8	1.06 to 21.9	36.7 to 81.3
Both areas combined (n = 50)	Mean	37.7	2.8	16.7	7.0	66.4
	SD	23.4	2.8	5.3	3.6	28.9
	Median	34.4	2.0	17.3	5.5	66.4
	IQR	22.2	1.3	5.6	3.2	29.0
	Range	8.5 to 201.2	0.67 to 17.8	5.6 to 45.0	0.67 to 27.7	20.5 to 234.3

^1^Means, standard deviations and range of THM concentrations across LGAs for SWC and HWC.

^2^SWC: 18.7% of values were below the detection limit in 1995–1998 and 63.6% from 1999 onwards. HWC: 51% of values were below the detection limit.

^3^HWC data available from 1997 to 2001.

The results of our statistical modeling of CRC incidence with THM concentrations at the LGA level are summarized in Table 2. We expressed the results as the incidence rate ratio (IRR) of CRC for an IQR increase in THM species. Using five year lag of exposure, for individual THM species, there was a positive association between bromoform concentration and incidence of CRC in men with IRR = 1.025 (95% CI 1.01, 1.040), but not in women. Analysis by cancer site showed that the positive association of CRC incidence in men with bromoform concentration was evident only for colon cancer. The IRRs for rectal cancer were not significantly increased for men or women for total THM or any of the THM species. All other site-specific associations were close to the null.

**Table 2:** **Incidence rate ratio of colorectal, colon and rectal cancer in men and women, lagged by 5 years, per IQR**^**1**^ **increase in total THMs and THM species, adjusted for socio-economic status, area of residence, year of incidence, water source**^**2**^**, smoking**^**3**^**, risky alcohol consumption**^**4 **^**and spatial random effect**

**THM species**	**Sex**	**Colorectal cancer**	**Colon cancer**	**Rectal cancer**
Total THM	Male	1.018 (0.995, 1.040)	1.020 (0.990, 1.049)	1.013 (0.977, 1.049)
Chloroform	Male	1.010 (0.988, 1.032)	1.010 (0.983, 1.037)	1.011 (0.980, 1.045)
Bromoform	Male	1.025 (1.009, 1.040)	1.035 (1.017, 1.053)	1.011 (0.989, 1.036)
BDCM	Male	1.018 (0.994, 1.044)	1.024 (0.994, 1.055)	1.012 (0.972, 1.051)
DBCM	Male	1.010 (0.984, 1.035)	1.017 (0.987, 1.050)	0.996 (0.959, 1.037)
Total THM	Female	0.977 (0.953, 1.001)	0.976 (0.946, 1.006)	0.984 (0.940, 1.027)
Chloroform	Female	0.978 (0.954, 0.999)	0.974 (0.946, 1.003)	0.989 (0.946, 1.038)
Bromoform	Female	1.003 (0.987, 1.018)	0.999 (0.979, 1.019)	1.012 (0.982, 1.042)
BDCM	Female	0.979 (0.956, 1.000)	0.983 (0.952, 1.014)	0.973 (0.926, 1.024)
DBCM	Female	0.995 (0.970, 1.021)	0.997 (0.966, 1.028)	0.994 (0.945, 1.045)

^1^IQR: Total THM = 29 μg/L, Chloroform = 22 μg/L, Bromoform = 2 μg/L, BDCM = 6 μg/L and DBCM = 4 μg/L.

Note that the IQR values have been rounded to estimate IRR for ease of interpretation.

^2^Proportion of people using tap water in each LGA.

^3^Proportion smokers in each LGA.

^4^Proportion of risky alcohol drinker.

The adjusted IRR for year of diagnosis of CRC was below one for both sexes and for all species of THMs, indicating that CRC incidence rates were falling over the period of the study (not shown in the analysis). The adjusted IRR for the relative index of inequality were below unity for all exposure categories, more so in women than men. Overall, men in the lowest socioeconomic quintile had 7% less risk of CRC than men in the highest quintile; women in the lowest socioeconomic quintile had 12% less risk than women in the highest quintile (not shown in the analysis). Men whose drinking water came from sources other than tap water had 24% less risk and for men who engaged in high risk drinking there was a 29% higher risk of CRC but none of these associations was statistically significant.

Removing socio-economic status from the model, exclusion of HWC area data or removal of the spatial random effect did not have an influential effect on the IRRs for total THMs or individual THM species (Table 3). In particular, the significant association of CRC incidence in men with bromoform concentration persisted regardless of these changes. Testing for pure specification bias by adding the variances of the LGA mean THM concentrations to the models reduced the IRRs in men somewhat but had little effect on women (Table 3). When we repeated this analysis including only LGAs where the variance was available (87%), the results were not different from those including the missing variances (not shown in the analysis). Exclusion of the spatial random effect term from the model in Table 2 had no material effect on the IRRs for any THM species for either sex (Table 3). When we averaged the THM concentration across the calendar years 1995–2001, in place of the five-year lag, and re-fitted the Bayesian model, the positive associations became slightly stronger and the negative associations shifted towards the null with reduced precision. In particular, the association for CRC in men and bromoform concentration became much stronger (IRR = 1.064, 95% CI 1.021, 1.113).

**Table 3:** **Sensitivity analyses for the model of incidence rate ratios for colorectal cancer per IQR**^**1 **^**increase in concentration of total THMs and selected specific THM species after adjusting for the covariates mentioned in Table**2

**THM species**	**Sex**	**Average exposure**^**2**^	**IRSD excluded**	**Restricted to SWC area**	**Spatial random effect excluded**	**Variance of THMs added**
Total THM	Male	1.033 (0.954, 1.119)	1.018 (0.993, 1.041)	1.018 (0.993, 1.041)	1.019 (0.997, 1.042)	1.007 (0.979, 1.032)
Chloroform	Male	1.022 (0.946, 1.110)	1.008 (0.985, 1.031)	1.008 (0.985, 1.031)	1.011 (0.990, 1.034)	1.005 (0.982, 1.029)
Bromoform	Male	1.064 (1.021, 1.113)	1.025 (1.011, 1.040)	1.025 (1.011, 1.040)	1.026 (1.011, 1.041)	1.020 (1.005, 1.036)
BDCM	Male	1.031 (0.973, 1.102)	1.022 (0.995, 1.050)	1.022 (0.995, 1.050)	1.021 (0.996, 1.046)	1.008 (0.980, 1.039)
DBCM	Male	0.998 (0.956, 1.042)	1.028 (0.999, 1.058)	1.028 (0.999, 1.058)	1.012 (0.988, 1.038)	1.000 (0.975, 1.029)
Total THM	Female	0.986 (0.919, 1.056)	0.973 (0.948, 0.999)	0.973 (0.948, 0.999)	0.980 (0.957, 1.004)	0.970 (0.945, 0.994)
Chloroform	Female	1.004 (0.930, 1.087)	0.974 (0.950, 0.997)	0.974 (0.950, 0.997)	0.980 (0.957, 1.004)	0.979 (0.951, 1.006)
Bromoform	Female	1.008 (0.965, 1.050)	1.005 (0.989, 1.023)	1.005 (0.989, 1.023)	1.004 (0.987, 1.020)	1.005 (0.985, 1.023)
BDCM	Female	0.992 (0.937, 1.045)	0.976 (0.948, 1.005)	0.976 (0.948, 1.005)	0.981 (0.957, 1.006)	0.973 (0.938, 1.007)
DBCM	Female	0.982 (0.942, 1.020)	0.997 (0.964, 1.028)	0.997 (0.964, 1.028)	0.995 (0.971, 1.021)	0.995 (0.966, 1.023)

^1^IQR: Total THM = 29 _g/L, Chloroform = 22 _g/L, Bromoform = 2 _g/L, BDCM = 6 _g/L and DBCM = 4 _g/L.

Note that the IQR values have been rounded to estimate IRR for ease of interpretation.

^2^Exposure averaged for all relevant years of the study.

## Discussion

We found a positive association between CRC incidence in men lagged five years, with bromoform concentrations in water. This association was largely confined to colon cancer. There was no appreciable association of colorectal cancer with any other species of THMs or with total THMs for either sex. The results showed little sensitivity to removal of SES (IRSD), the spatial random effect term, or addition of the within-area THM variance to the model. When the exposure was averaged across the calendar years, in place of the five-year lag, the positive associations became slightly stronger with reduced precision, possibly because of less attenuation due to non-differential measurement error.

The ecological study design is a primary limitation of our study because we could not adjust adequately for individual level covariates such as consumption of meat and vegetables, which might confound the association of CRC with THM concentrations. The ecological study design also did not allow us to incorporate estimated exposures relevant to the etiologic period that might be gleaned from long-term residential and drinking water histories. That we used only five years of lag between exposure and outcome is another limitation; other investigators have argued that even a 34 year period between the beginning of exposure and the end of follow-up might be too short to detect an effect of environmental exposure on mortality from cancer [27]. However, available evidence on THMs suggests that they may cause cancer by gene mutation or by cell proliferation followed by cytotoxicity, with the former more prominent for the brominated species [28-30]. Thus it is possible that effects could be seen after both short and long periods. In our study the THM exposure data required for a long lag period were not available (available only from 1995). Another major limitation to our study was that we made no assumption about contribution of THMs from different exposure routes (inhalation and dermal absorption through showering, bathing or swimming or other water use activities) which may be significant contributors to the overall exposure to THMs [31]. The associations observed may not be directly related to THMs but with unmeasured DBPs correlated with their formation [32].

A further weakness of the ecological design is that people often change their residence and hence their exposure status, if DBP concentration varies spatially (which was true in our case and necessary to the ecological design). Australian Census data for 2007 show that 80% of people in NSW did not change their residential address in the previous year, 6% moved to another address in the same Statistical Local Area (SLA) (SLAs are the same as or, for a small number of cases, subsets of LGAs), and 14% moved a greater distance [33]. For the previous five years, 55% of people did not change their residential address, 13% moved to another address within the same SLA and 32% moved a greater distance [34]. We did not take into account the exposure misclassification which might have resulted from this population mobility.

The main strengths of our study are the use of estimated annual mean concentrations of THM species for small areas, albeit often with missing data; a lag period between exposure and outcome; and more advanced statistical methods. No previous study of this type has used exposure data at this level of detail or specificity (monthly measurement of THMs), or used a lag period in the analysis [35-40]. Annual exposure estimates and disease rates, and a lag period may be quite important because THM concentrations in water vary over time due to factors such as changes in disinfection practice (e.g., change from chlorination to chloramination and change in the chlorine to ammonia ratio), installation of filters in water treatment plants, rechlorination practices throughout the distribution systems and change in environmental conditions (drought breaking rains triggered the increase in brominated trihalomethanes and chloroform concentration around 1998) [9].

Another strength are the analytical methods used which included Poisson variability, spatial random effects and within-area variability of exposure that permitted control for pure specification bias. Previous ecological analyses of associations of DBPs with cancer risk have used only correlation coefficients or simple linear regression. Correlation coefficients mix the strength (size) and precision of an association, whereas the regression coefficient reports them separately. Simple linear regression using rate as the outcome is prone to several problems. First, the assumption of homoscedasticity (that is, the error term of the regression model has a constant standard deviation that does not depend on the value of the outcome) is a particular issue for studies that have used unweighted linear regression. Generally, small areas have unstable rates and including them in a regression model without taking account of this factor can cause unpredictable bias. Second, simple linear regression assumes independence of spatial units, such that there would be no clustering of adjacent areas with similar rates, which is unlikely. Third, least squares estimators assume that the rates or log of the rates are normally distributed, whereas we would generally expect rates in small areas to have a Poisson distribution. The analytical methods we have used address all of these issues; thus our results are more likely to faithfully reflect the reality than results based on linear regression. In addition, our methods have allowed us to largely rule out within area variability and spatial autocorrelation as explanations for the positive associations we observed between bromoform concentrations and colon cancer.

To our knowledge, this is the first study in which the associations between colon and rectal cancers and THM species concentrations in drinking water have been examined. The finding of a relationship between bromoform and colorectal cancer might be dismissed as being a chance finding, given the multiple testing. However, some recent results suggest otherwise. In a case–control study of rectal cancer in men, Bove and co-workers [1] found a positive association of rectal cancer with bromoform (OR 1.20, 95% CI 1.05, 1.35). Like us, Bove and co-workers [1] found no association of rectal cancer with chloroform or total THMs. While it is possible that the lack of an association between rectal cancer incidence and bromoform in our study could be due to the short lag period, since ‘low-level’ environmental exposures may require several decades to cause a detectable effect on cancer risk [27,41], it might also be due to lack of statistical power, since we did find an association with colon cancer. The only other study of the association of colon or rectal cancers with a specific THM found a positive association of chloroform with colon cancer (RR 1.68, 95% CI 1.11, 2.53), but not rectal cancer (RR 1.07, 95% CI 0.60, 1.93), in postmenopausal women; other species of THMs were not examined [42]. We found no evidence of such an association in either men or women.

Our recent meta-analysis of cohort and case–control studies of total THM exposure and colon and rectal cancers [8] found that risks of both were increased in the highest category of exposure relative to the lowest: colon cancer OR 1.33 (95% CI 1.12, 1.57) and rectal cancer OR 1.30 (95% CI 1.06, 1.59). These results suggest that THMs may increase the risk of both colon and rectal cancer and that if the effect is specific to a particular species of THM that species is a large component of the total or it has a strong effect. The former, at least, is not true for bromoform [28,43]. There were too few sex-specific results to see if there were risk differences by sex of colon or rectal cancers with THM exposure in the meta-analysis.

Expert evaluation of the evidence has concluded there is limited evidence for the carcinogenicity of bromoform in experimental animals. This evaluation, however, was made in 1999 and was based on only one relevant experimental study [29]. With respect to the other THM species, there was considered to be sufficient evidence for the carcinogenicity of chloroform [44] and bromodichloromethane [45] in experimental animals, when evaluated in 2005, but only limited evidence for dibromochloromethane [46] (evaluated in 1999). Some individual studies, however, have found that brominated trihalomethanes (bromoform, bromodichloromethane and dibromochloromethane) are more carcinogenic than chloroform [47,48]. In our study, both bromodichloromethane and dibromochlormethane were more strongly positively associated with colorectal cancer, or just colon cancer in men, than were chloroform or total THMs. The IRRs for all brominated trihalomethanes in men were 1.072 (95% CI 1.033, 1.113) for colon cancer and 1.080 (95% CI 1.031, 1.131) for rectal cancer (not shown in the analysis). Considered together the experimental and epidemiological results suggest that further investigation of the associations of brominated trihalomethanes with colon and rectal cancers is warranted.

While causality between THM exposure and colorectal cancer has not been established, most people in developed countries, drink water containing THMs. Therefore, a small association projected across the globe could translate into a large number of cases. Thus the reason the associations between THMs, and particularly bromoform, and colon and rectal cancers should be investigated further in higher quality epidemiological studies.

Since the existing epidemiological studies provide some, although weak evidence, that the effects of different THM species could differ by sex and cancer site, future studies should measure individual species of THMs and study the effects on men and women with regard to colon and rectal cancers separately. The studies should be sufficiently powered to detect small associations, as that small association could translate into an important number of cases.

## Conclusion

We found weak evidence of a positive association between incidence of colorectal cancer in men and total THM concentration in water supplies and a somewhat stronger association for bromoform concentration. This was primarily because of an association of colon cancer incidence in men and bromoform concentration. Although an association of rectal cancer in men with bromoform concentration has been observed previously, the association we observed with colon cancer has not been previously reported. The inconsistency of these observations by cancer type and the ecological study design prevent inference of causation. However, the potential population impact of such an association justifies further research into the effects of THMs in drinking water, particularly the brominated species, and colorectal cancer.

## Abbreviations

ABS: Australian Bureau of Statistics; CRC: Colorectal cancer; DBP: Disinfection by-products; DIC: Deviance information criterion; DL: Detection limit; HWC: Hunter Water Corporation; IQR: Inter-quartile range; IRR: Incidence rate ratio; IRSD: Index of relative socio-economic disadvantage; LGA: Local government area; MCMC: Markov Chain Monte Carlo; NSW: New South Wales; SEIFA: Socio-Economic Indexes for Areas; SES: Socio-Economic Status; SLA: Statistical Local Area; SWC: Sydney Water Corporation; THM: Trihalomethane.

## Competing interests

T Driscoll, C Cowie and B Armstrong were recipients of a contract from the Sydney Water Corporation to study the health of sewer workers and MB Rahman worked on the contract. Sydney Water Corporation has assisted their research by giving them access to results of routine measurements of water quality, including concentrations of disinfection by-products.

## Authors’ contributions

BR conceived the study, conducted the analyses and drafted the manuscript. CC conceived the study and contributed drafting the manuscript. TD conceived the study and proof read the manuscript. RS collected and collated some of the information for the study and contributed drafting some parts of the manuscript. BA conceived, designed and planned the study, help conducting the study and revised the manuscript. MC supervised all the analyses and conducted some of the analyses and proof read the manuscript. All authors read and approved the final manuscript.

## Authors’ information

Institution where the work was done: School of Public Health, The University of Sydney, NSW 2006, Australia.

## Pre-publication history

The pre-publication history for this paper can be accessed here:

http://www.biomedcentral.com/1471-2407/14/445/prepub

## Supplementary Material

Additional file 1 SWC Distribution Systems and LGA coverage. Click here for file

Additional file 2 Sensitivity analysis of values below the detection limit. Click here for file
