# Supplementary figures and images for: Colon and rectal cancer incidence and water trihalomethane concentrations in New South Wales, Australia
Source: BMC Cancer. 2014 Jun 17;14:445. doi: 10.1186/1471-2407-14-445 (PMC4088985; doi:10.1186/1471-2407-14-445)

Additional file

**
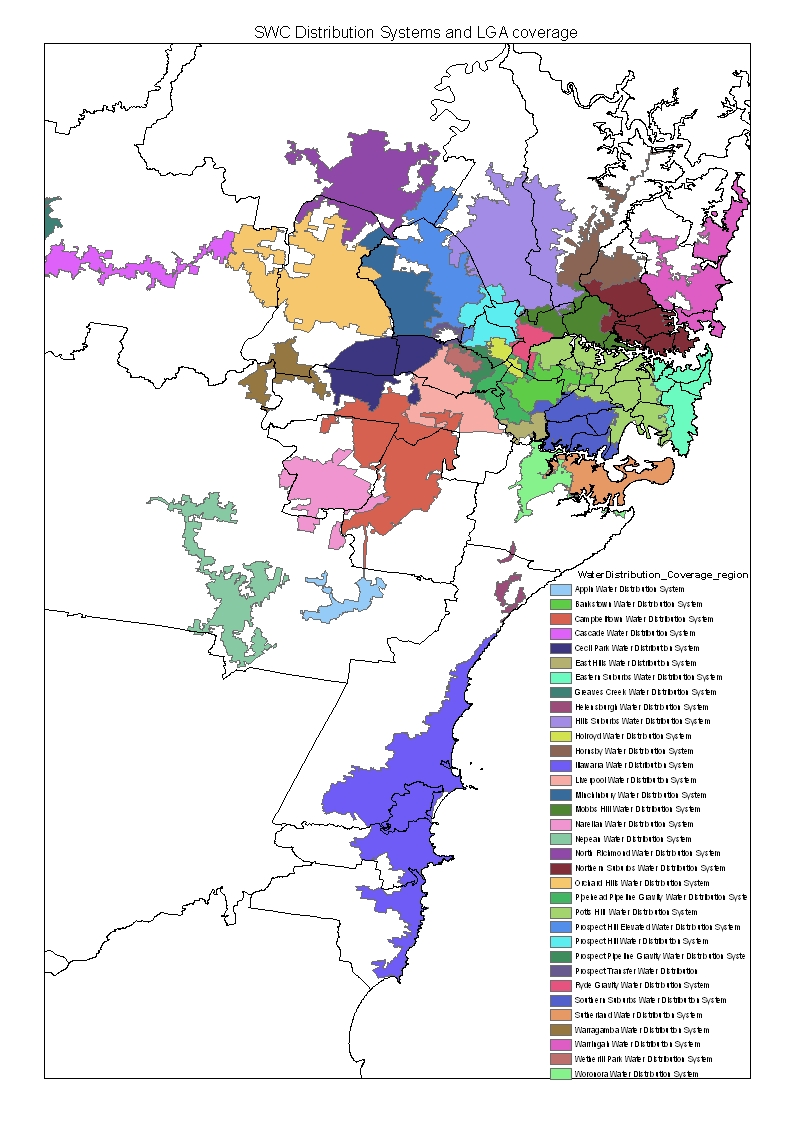
**

Supplement: Additional file 1 — SWC Distribution Systems and LGA coverage. [file 1471-2407-14-445-S1.docx]
