# Supplementary material for: Colon and rectal cancer incidence and water trihalomethane concentrations in New South Wales, Australia
Source: BMC Cancer. 2014 Jun 17;14:445. doi: 10.1186/1471-2407-14-445 (PMC4088985; doi:10.1186/1471-2407-14-445)
Supplement: Additional file 2 — Sensitivity analysis of values below the detection limit. [file 1471-2407-14-445-S2.doc]

**Sensitivity analysis of values below the detection limit**

In the study values below detection limit for different species of THM was an important issue. We replaced values below the DL with a value that was two thirds of the DL. Because there were many values below the DL for bromoform we also undertook a sensitivity analysis, using two other methods for replacing the DL values. In the first method, all the DL values were replaced with zero. In the second method, we first estimated the proportion of values reported as less than three µg/L that were less than one µg/L using data collected in the period when the DL was less than one. This proportion of the values reported as less than three µg/L for the whole study period were considered to be less than one µg/L and substituted with 0.667. The remaining values less than three µg/L (which were presumed to be between one and less than three) were replaced with values estimated from the corresponding dibromochloromethane (DBCM) values, as the bromoform values were observed to be positively correlated to the DBCM values for 1995 to 1998. This third method was applicable to only the SWC data.

The difference in bromoform concentrations was sensitive to the values substituted for readings below the DL. When zero was substituted instead of two thirds of the DL for readings below the DL, the mean values in Table 1, fell for SWC from 2.86µg/L to 1.71µg/L and for HWC from 1.76µg/L to 1.31µg/L. A third method applicable to SWC only (as described previously) gave a mean bromoform concentration of 2.39 µg/L.
